# Reevaluating Dengue Management: Insights Into Fever Trends, Thrombocytopenia, and Clinical Outcomes

**DOI:** 10.7759/cureus.81736

**Published:** 2025-04-04

**Authors:** Usman Zafar, Ashir Iqbal, Farrukh Ansar, Abdullah Azzam, Moiz N Butt, Sundas N Butt, Junaid Ahsan, Muhammad A Awan, Swaiza Pervaiz, Kanza Mubasher

**Affiliations:** 1 Department of Internal Medicine, Alkhidmat Raazi Hospital, Rawalpindi, PAK; 2 Department of Medicine, Alkhidmat Raazi Hospital, Rawalpindi, PAK; 3 General Surgery, Dr. Akbar Niazi Teaching Hospital, Islamabad, PAK; 4 Medicine, Aziz Bhatti Shaheed Teaching Hospital, Gujrat, PAK

**Keywords:** dengue fever, home-based care, hospital admissions, platelet trends, thrombocytopenia

## Abstract

Introduction

Dengue fever remains a major public health challenge in endemic regions, leading to significant hospital admissions and resource utilization. This study aimed to analyze the clinical course of dengue, focusing on fever trends, thrombocytopenia, hematocrit fluctuations, and bleeding manifestations.

Methods

A retrospective observational study was conducted on 135 confirmed dengue patients admitted to Alkhidmat Raazi Hospital, a secondary care hospital in Rawalpindi, which is a dengue-endemic region. Clinical and laboratory data, including fever patterns, platelet counts, hematocrit levels, and total leukocyte count trends, were recorded over the course of the illness. Patient outcomes and symptomatology were analyzed to determine the necessity of hospitalization.

Results

Among 135 patients, 118 (87.4%) had uncomplicated dengue fever, while 12 (8.9%) developed dengue hemorrhagic fever. Despite all patients experiencing thrombocytopenia at some stage, 19 (14.1%) had platelet counts below 20,000/µL without any bleeding. Fever trends followed a biphasic pattern, with resolution observed in 101 (74.5%) patients by day 10. A total of 38 (28.1%) patients remained afebrile for over 72 hours despite a declining platelet count, indicating fever resolution independent of thrombocytopenia. The median length of hospital stay was three days (range: 1-10 days), with 107 (79.3%) patients having no comorbidities. Bleeding manifestations were seen in 22 (16.3%) patients, with nasal bleeding in eight (36.3%) and gum bleeding in six (27.2%) patients.

Conclusion

This study reinforces the idea that hospital admissions for dengue should not be based solely on platelet counts. Structured outpatient monitoring could help reduce unnecessary admissions, optimizing healthcare resources. Future studies should explore home-based care models to improve dengue management.

## Introduction

Dengue fever is a major global public health concern, particularly in tropical and subtropical regions, where the disease burden continues to rise [1]. Dengue virus (DENV), a mosquito-borne flavivirus, is transmitted primarily by *Aedes aegypti *and *Aedes albopictus* mosquitoes [1]. The World Health Organization (WHO) estimates that over 500 million dengue infections occur annually, with severe cases leading to significant morbidity and mortality [2]. The clinical spectrum of dengue fever varies widely, ranging from self-limiting febrile illness to severe complications such as dengue hemorrhagic fever (DHF) and dengue shock syndrome (DSS) [3]. Despite advancements in diagnostic methods and supportive care, dengue-related hospitalizations remain a significant burden on healthcare systems, particularly in endemic countries like Pakistan [4].

The clinical and laboratory progression of dengue fever follows a characteristic pattern. The disease typically begins with a febrile phase, characterized by high-grade fever, myalgia, headache, retro-orbital pain, and leukopenia [5]. This phase is followed by the critical phase (days 4-7), during which patients may develop plasma leakage, thrombocytopenia, and hemoconcentration, increasing the risk of complications such as shock and bleeding [5]. The recovery phase is marked by gradual normalization of platelet counts, resolution of fever, and stabilization of hematocrit levels [5]. However, the timing and severity of these trends may vary, necessitating close monitoring and individualized management strategies to prevent complications [5].

Several studies have attempted to characterize the hematological and clinical trends in hospitalized dengue patients, but variations in disease course, regional differences, and population-specific patterns remain underexplored [6]. In Pakistan, where dengue outbreaks are recurrent, there is a need for local data on disease progression, which can aid in refining triage strategies, risk stratification, and treatment protocols [7]. A better understanding of platelet dynamics, hematocrit fluctuations, and fever resolution trends can provide valuable insights into critical illness phases, enabling clinicians to predict and manage severe cases more effectively [8].

This study aims to analyze the clinical and laboratory trends in hospitalized dengue patients, focusing on platelet counts, hematocrit levels, total leukocyte counts (TLC), fever trends, and associated complications. The primary objective of this study is to identify critical time points in the progression of dengue fever where patients are at the highest risk of complications, particularly thrombocytopenia, plasma leakage, and prolonged fever. The secondary objectives include evaluating the relationship between fever resolution and hematological trends, characterizing patterns of severe dengue cases, and identifying predictors of prolonged hospitalization or intensive care unit admission.

By systematically analyzing these parameters, this study aims to contribute to the growing body of knowledge on dengue pathophysiology and help improve early warning systems. The findings also aim to support improved clinical decision-making and more efficient resource allocation by informing hospital admission practices and guiding policy development during dengue outbreaks.

## Materials and methods

Study design and setting

This cross-sectional study was conducted at Alkhidmat Raazi Hospital, Rawalpindi, Pakistan, a secondary care hospital that manages a high volume of dengue fever cases during seasonal outbreaks. The study included all dengue cases admitted to the hospital in 2024, ensuring a comprehensive analysis of hospitalized patients with confirmed dengue infection. A cross-sectional design with retrospective data collection was chosen to efficiently analyze a large dataset over a defined period, allowing for the identification of clinical trends and outcomes during a peak outbreak season. Data were collected retrospectively from patient medical records from 2024.

Study population and eligibility criteria

The study included a total of 135 patients who were admitted to the hospital with a confirmed diagnosis of dengue fever. The inclusion criteria were strictly limited to inpatients with laboratory-confirmed dengue, diagnosed either through NS1 antigen testing or serological confirmation via IgM and IgG ELISA. Patients managed exclusively in the outpatient department and those without confirmed laboratory results were excluded from the study. Outpatient data were excluded due to potential variability in follow-up, limited clinical documentation, and less reliable outcome assessment, which could compromise the consistency and accuracy required for analyzing clinical progression and complications.

Data collection

A structured and standardized data collection process was implemented to ensure accuracy and minimize data entry errors. Patient medical records and electronic health system data were systematically retrieved and reviewed. To facilitate comprehensive data management, a dedicated Excel spreadsheet was developed with predefined fields to capture all relevant information. The spreadsheet was designed to include key categories such as demographics, laboratory parameters, clinical presentation, disease progression, complications, radiological findings, and patient outcomes. Demographic data encompassed variables such as age, gender, comorbidities, and admission date. Laboratory parameters included platelet counts, hematocrit levels, leukocyte counts, and other relevant biomarkers. Clinical parameters recorded were symptoms at presentation, fever duration, warning signs, and any bleeding manifestations. The day of admission in relation to the illness course was documented to assess trends in disease progression. Additionally, complications such as severe dengue, organ involvement, and the need for intensive care were identified. Radiological findings, including ultrasound evidence of ascites or pleural effusion and chest X-ray abnormalities, were also incorporated. Outcome measures focused on recovery, hospital stay duration, discharge status, and any reported mortality.

To enhance data accuracy and reliability, the extraction process was independently performed by two separate teams, each consisting of two trained data extractors. Both teams reviewed the same set of medical records and entered the extracted information into the structured Excel sheet. Any discrepancies between the two datasets were identified and subsequently reviewed by a senior author, who resolved inconsistencies through consensus. This rigorous validation process significantly minimized inter-observer variability and ensured a high level of data reliability, thereby strengthening the study’s methodological integrity.

Data entry and statistical analysis

The finalized dataset was entered into IBM SPSS Statistics for Windows, version 26.0 (released 2019, IBM Corp., Armonk, NY) for statistical analysis. Quality control measures included double data entry verification to prevent transcription errors. Descriptive statistics were used to summarize continuous variables, with mean, standard deviation (SD), and median, where applicable. Categorical variables were presented as frequencies and percentages. Statistical comparisons between subgroups were performed using chi-square tests for categorical variables and t-tests or Mann-Whitney U tests for continuous variables, depending on data normality.

Ethical considerations

This study was conducted in accordance with institutional ethical guidelines and the principles outlined in the Declaration of Helsinki. As this was a retrospective study utilizing anonymized patient data, the requirement for individual patient consent was waived. All patient records were de-identified prior to data extraction, ensuring strict confidentiality and anonymity throughout the research process. No personally identifiable information was recorded or disclosed at any stage of data collection, analysis, or reporting.

The study protocol was reviewed and approved by the Institutional Review Board of Alkhidmat Raazi Hospital, Rawalpindi, Pakistan (approval no. IRB/A/01/24). All collected data were securely stored and accessed only by authorized researchers, strictly adhering to data protection and privacy regulations.

## Results

Demographic and clinical characteristics

The study included 135 patients with a mean age of 40.31 years (SD: 15.72, range: 15-75 years). All patients were presented in the months of August to November 2024. There were 93 (68.89%) males and 42 (31.11%) female patients. The majority of cases were diagnosed as DF (118, 87.4%), with 13 (9.62%) cases classified as DHF. In addition, three (2.2%) patients presented with DF complicated by lower respiratory tract infection (LRTI), while one (0.7%) patient developed expanded dengue syndrome. Diagnosis was confirmed primarily through NS1 antigen detection in 116 (85.9%) cases, with 19 (14.1%) cases identified via dengue serology (IgM antibodies against dengue). A total of 107 (79.3%) patients had no known comorbidities, while 18 (13.3%) had hypertension and diabetes mellitus, all of whom recovered without complications. The study included five elderly patients (3.7%) and three pregnant women (2.2%), with no adverse outcomes reported in these subgroups. The mean length of hospital stay (LoS) was 3.65 days (range: one to 10 days, median: 3.0 days), with 25th percentile at 2.5 days and 75th percentile at four days. Table 1 shows the baseline characteristics of the study population.

**Table 1:** Baseline characteristics of the study population

Characteristic	N = 135	Percentage (%)
Gender		
Male	93	68.89%
Female	42	31.11%
Dengue diagnosis		
Dengue fever	118	87.4%
Dengue hemorrhagic fever	13	9.62%
Dengue with LRTI	3	2.2%
Expanded dengue syndrome	1	0.7%
Diagnosis method		
NS1	116	85.9%
Dengue serology (IgM antibodies against dengue)	19	14.1%
Patient outcomes		
Recovered & discharged	129	95.5%
Discharge on request	5	3.7%
Referred	1	0.7%
Imaging findings		
Ultrasound (ascites/pleural effusion)	21	15.5%
Chest X-ray (effusion)	1	0.7%
Comorbidities		
None	107	79.3%
Hypertension + diabetes mellitus	18	13.3%
Elderly	5	3.7%
Pregnancy	3	2.2%

Symptomatology

The most commonly presenting symptoms were constitutional and musculoskeletal in nature, with myalgia reported in 112 cases (82.9%), followed by headache in 89 cases (65.9%); arthralgia, backache, or bone pain in 50 cases (37.0%); and retro-orbital pain in 35 cases (25.9%). Less frequent constitutional symptoms included lethargy and restlessness in three cases (2.2%) and giddiness in one case (0.7%). Gastrointestinal symptoms were also observed, including persistent vomiting in 17 cases (12.6%) and abdominal pain in 13 cases (9.6%). Decreased urine output was reported in two cases (1.5%). Cutaneous manifestations, such as rash, were documented in eight cases (5.9%), while bleeding manifestations were seen in six cases (4.4%).

Febrile versus afebrile trends

**Figure 1:**
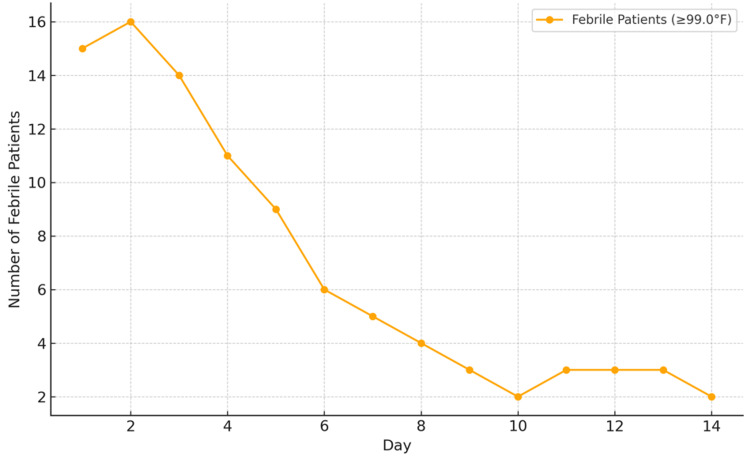
Fever resolution trend in dengue patients over the course of illness

The analysis of febrile (≥99°F) and afebrile (<99°F) patients shows a variable duration among patients from one to 14 days. On day 1, 75% of the patients presented with fever, while 25% were afebrile. This trend intensified on day 2, where febrile cases peaked at 80%, making it the most fever-dominant day of illness. However, by day 3 and day 4, the proportion of febrile patients declined to 70% and 55%, respectively, marking the beginning of a transition phase. Figure 1 shows the fever resolution trend in dengue patients over the course of illness.

Platelet trends and hematological findings

**Figure 2:**
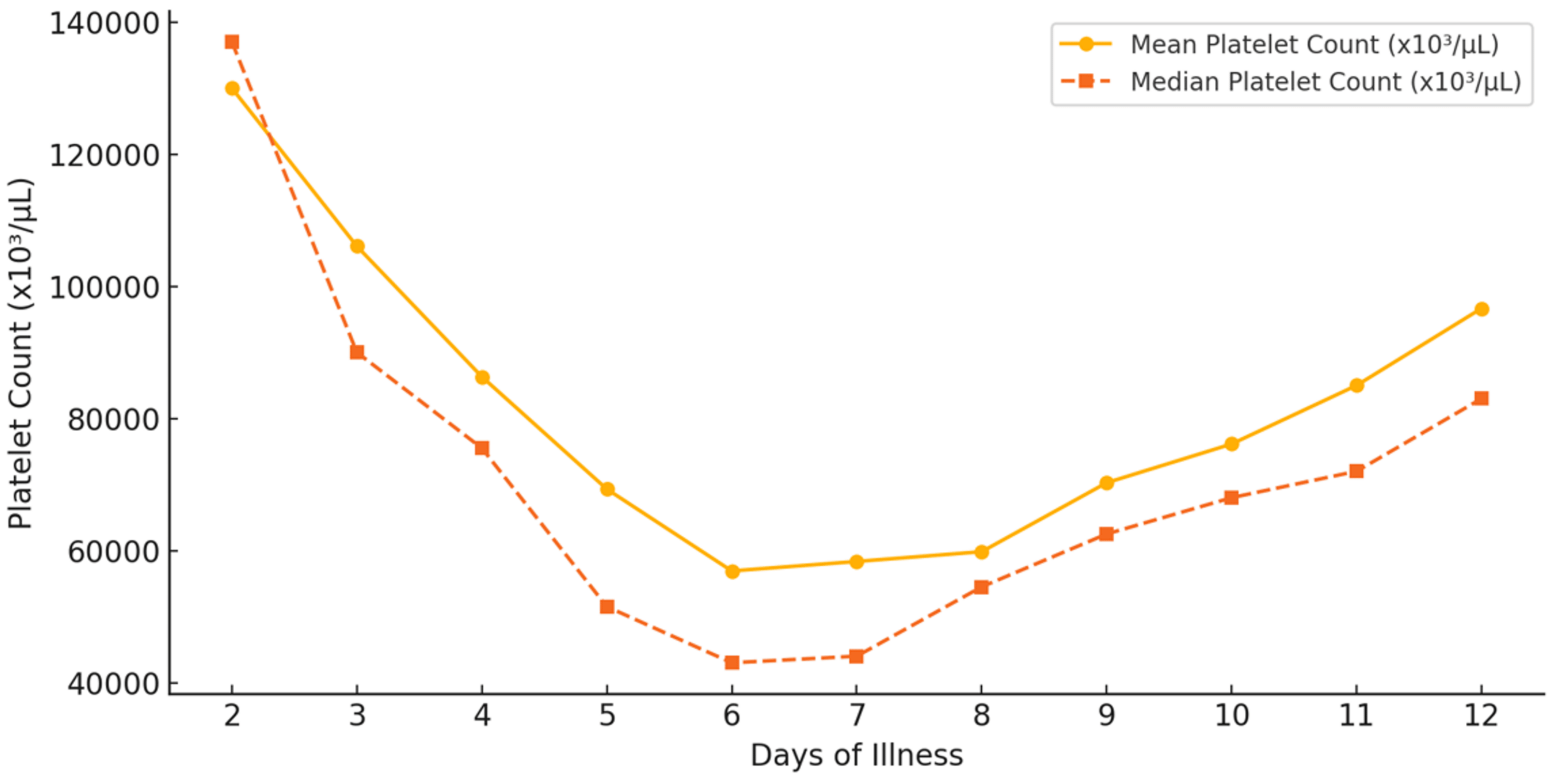
Platelet count trends in dengue patients over time

All patients developed thrombocytopenia (<150,000/µL) at some stage of illness, with 16 patients exhibiting normal platelet counts upon admission before a subsequent decline. Figure 2 shows the platelet trend over time (days of illness). A downward trajectory in platelet levels was seen, with a mean platelet count of 130,000/µL on day 3, which progressively decreased to 106,074/µL on day 4, 86,312/µL on day 5, and 69,300/µL on day 6. The nadir occurred between days 6 and 8, with the lowest median platelet count of 43,000/µL recorded on day 7 (mean: 56,906/µL). The lowest recorded platelet count was 6,000/µL. Platelet recovery commenced around day 9 (mean: 59,820/µL) and approached the 100,000/µL threshold by days 12 and 13. Table 2 shows the detailed values of platelet count over time.

**Table 2:** Platelet count trends over the course of illness in dengue patients

Day of illness	Mean (µL)	Median (µL)	Min (µL)	Max (µL)	Number of patients
Day 3	130000	137000	34000	246000	11
Day 4	106074	90000	29000	223000	27
Day 5	86312	75500	7000	281000	48
Day 6	69300	51500	8000	190000	80
Day 7	56906	43000	10000	216000	85
Day 8	58341	44000	11000	188000	85
Day 9	59820	54500	9000	169000	78
Day 10	70260	62500	7000	224000	50
Day 11	76129	68000	6000	182000	31
Day 12	85000	72000	16000	188000	17
Day 13	96667	83000	45000	231000	6

Hematocrit variability

The hematocrit (HCT) trend shows an initial rise with a gradual decline. The early phase (days 2-4) exhibited stable hematocrit values, with a mean HCT of 39.03% on day 2 and 36.45% on day 3, suggesting minimal hemoconcentration. By day 4, the mean hematocrit increased to 40.78%, reaching a maximum of 49.6%, marking the onset of plasma leakage. The critical phase (days 5-8) showed the highest hemoconcentration, with day 5 recording the peak mean HCT (42.03%) and a maximum of 61.0%. Recovery ensued from day 9, with the mean HCT decreasing to 40.33%, stabilizing around 39.0% by days 12-13, indicating the resolution of plasma leakage.

**Figure 3:**
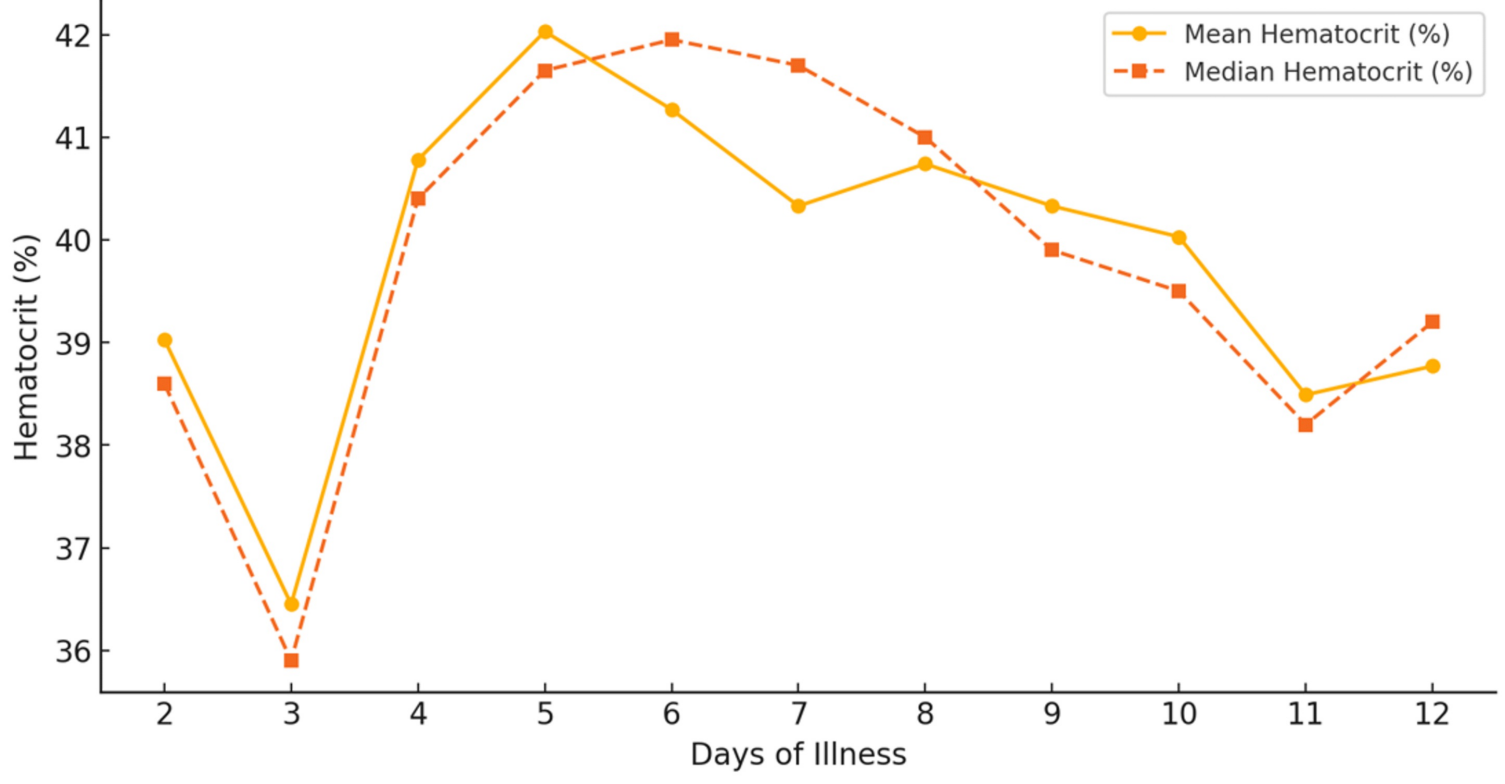
Hematocrit count trends in dengue patients over time

**Table 3:** Hematocrit trends over the course of illness in dengue patients

Day of illness	Mean HCT (%)	Median HCT (%)	Min HCT (%)	Max HCT (%)	Number of patients
Day 2	39.03	38.6	35.4	43.5	4
Day 3	36.45	35.9	34.2	41.1	6
Day 4	40.78	40.4	31.8	49.6	27
Day 5	42.03	41.65	30.3	61.0	46
Day 6	41.27	41.95	14.5	56.3	78
Day 7	40.33	41.7	11.9	53.4	83
Day 8	40.74	41.0	22.0	55.0	83
Day 9	40.33	39.9	21.0	52.1	74
Day 10	40.03	39.5	22.4	57.0	49
Day 11	38.49	38.2	22.6	53.1	31
Day 12	38.77	39.2	23.1	49.9	17
Day 13	39.0	39.1	27.2	47.4	6

Total leukocyte count (TLC) trends

The total leukocyte count (TLC) in dengue patients follows an initial decline during the febrile phase, followed by a gradual increase during recovery. Leukopenia, defined as a TLC below 4,000 cells/µL, was a common finding during the early phase of illness, particularly between days 4 and 6, where the lowest recorded median TLC was 3,575 cells/µL on day 5. The mean TLC remained below 5,000 cells/µL from days 2 to 7, indicating a suppressed immune response during the acute viral phase of the infection. As the disease progressed, a gradual recovery in leukocyte count was observed from day 8 onward, with the mean TLC increasing to 6,045 cells/µL on day 8 and further to 6,478 cells/µL on day 10. By day 13, TLC levels indicated a return to near-normal immune function. The data also revealed cases of leukocytosis (TLC > 11,000 cells/µL), particularly after day 5 (N = 6), potentially indicating secondary bacterial infections or an exaggerated inflammatory response.

**Figure 4:**
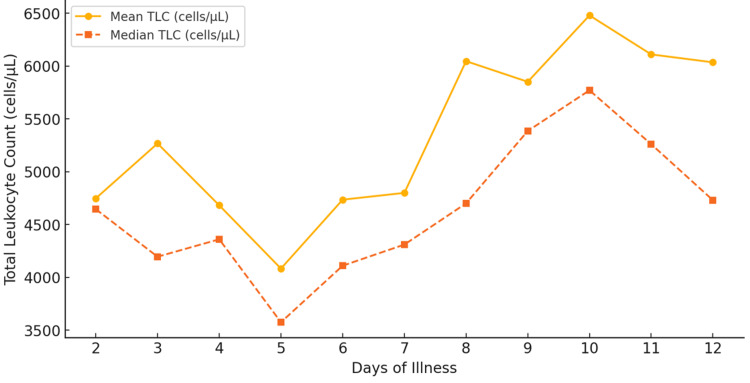
Total leukocyte count trends in dengue patients over time

**Table 4:** Total leukocyte count trends over the course of illness in dengue patients

Day of illness	Mean TLC (cells/µL)	Median TLC (cells/µL)	Min TLC (cells/µL)	Max TLC (cells/µL)	Number of patients
Day 2	4747.5	4645.0	3030.0	6670.0	4
Day 3	5266.7	4195.0	2970.0	9920.0	6
Day 4	4681.5	4360.0	1930.0	9110.0	27
Day 5	4083.0	3575.0	1690.0	13100.0	46
Day 6	4734.8	4110.0	1200.0	15800.0	80
Day 7	4799.1	4310.0	1840.0	10640.0	83
Day 8	6045.7	4700.0	1800.0	7180.0	83
Day 9	5850.4	5385.0	1990.0	17720.0	74
Day 10	6478.9	5770.0	720.0	17990.0	49
Day 11	6109.7	5260.0	2680.0	14750.0	31
Day 12	6034.7	4730.0	3600.0	11800.0	17
Day 13	7768.0	7800.0	4940.0	9640.0	6

Bleeding manifestations and their relationship with platelet trends

Among the sample population, 22 (16.3%) patients showed bleeding manifestation, with a mean platelet count of 56.57 ×10⁹/L (SD = 28.37 ×10⁹/L, range: 19.4 ×10⁹/L to 126.14 ×10⁹/L). The cohort consisted of individuals aged 18 to 65 years. Only two patients had HTN and diabetes, while 20 patients (90.9%) had no known comorbid conditions. Among the 22 patients, the most commonly observed hemorrhagic manifestation was nasal bleeding, which occurred in eight patients (36.3%). Gum bleeding was documented in six patients (27.2%). Other hemorrhagic presentations included hematuria in two patients (9.1%), petechiae in two patients (9.1%), melena in one patient (4.5%), menorrhagia in one patient (4.5%), and PR bleeding in one patient (4.5%). An analysis of the sequential platelet counts over multiple days revealed a progressive decline in platelet levels between days 2 and 5, followed by a gradual recovery phase in most cases. The lowest mean platelet count recorded was 19.4 × 10⁹/L in a patient with PR bleeding (age 48 years). In contrast, the highest mean platelet count of 126.14 ×10⁹/L was observed in a patient with petechial hemorrhage (age 32 years). Among all patients, 49 individuals (36.3%) had platelet counts below 50 × 10⁹/L at some point but did not develop any clinical bleeding. Furthermore, 19 patients (14.1%) had severe thrombocytopenia with platelet counts dropping below 20 × 10⁹/L without any bleeding symptoms. The lowest recorded platelet count was 6 ×10⁹/L, and this patient did not exhibit any hemorrhagic manifestations.

Trends observed

In this study, the progression of dengue illness followed a well-defined pattern, characterized by distinct early, critical, and recovery phases. The early phase (days 1-3) was marked by persistent fever and relatively stable platelet counts, with minimal changes in hematocrit and leukocyte levels. As the disease progressed into the critical phase (days 4-7), platelet counts showed a progressive decline, reaching their lowest levels between days 6 and 8. This coincided with a peak in hematocrit values, suggesting plasma leakage and fever resolution in most patients. Leukopenia was also most prominent during this period, reflecting immune suppression.

The recovery phase (days 8-13) was characterized by a gradual increase in platelet counts and a decline in hematocrit values toward normal levels. The overall convergence of nadirs in platelet counts, peak hematocrit levels, and fever resolution around days 5-7 highlights this period as the critical phase of illness, requiring the most intensive monitoring.

## Discussion

DF remains a major public health burden in endemic regions, with seasonal outbreaks leading to significant morbidity and healthcare resource utilization [9]. Our study highlights key trends in dengue progression, particularly regarding thrombocytopenia, bleeding manifestations, and hospital stay duration. A critical question that emerges from our findings is whether routine hospital admissions for dengue are always necessary. Given that most patients in our study had an uncomplicated course and that bleeding was rare even in cases of severe thrombocytopenia, we argue that hospital overcrowding due to dengue could be mitigated with more evidence-based admission criteria.

Our study’s findings align with global research on dengue, challenging the traditional emphasis on platelet count as a determinant for admission and care escalation [10]. The fear-driven approach, fueled by media sensationalism and misconceptions within the medical community, often leads to unnecessary hospital admissions, which strain healthcare resources and hinder the management of truly severe cases [11].

A central finding of our study was that all dengue patients developed thrombocytopenia at some stage, but only 16.3% exhibited bleeding manifestations, which were predominantly mild bleeds. Notably, even individuals with platelet counts as low as less than 20,000/µL did not develop significant hemorrhagic complications. These findings are consistent with previous studies that question the traditional emphasis on platelet counts as a predictor of bleeding risk [12].

A study from Singapore reported that among the DF cases studied, 788 patients experienced a platelet count drop below 20,000/mm³; however, none of them exhibited any clinical signs of bleeding [13]. Similarly, the literature suggests that spontaneous bleeding in dengue may be more closely related to platelet function and endothelial integrity rather than absolute platelet count alone [14]. Our findings align with these studies, further supporting the notion that thrombocytopenia, in isolation, should not be the sole determinant for hospital admission or platelet transfusion.

One of the most entrenched practices in dengue management is the liberal use of platelet transfusions for severe thrombocytopenia [15]. However, our study supports the growing body of evidence indicating that prophylactic platelet transfusions are often unnecessary. We observed that among the 14.1% of patients who had platelet counts below 20,000/µL, none developed life-threatening bleeding. This mirrors findings by Lee et al., who reported that prophylactic platelet transfusions did not significantly reduce bleeding risk in dengue patients [13]. In addition, a randomized controlled trial found that prophylactic platelet transfusions in DF did not improve clinical outcomes and were associated with complications such as fluid overload [16].

Updated literature review on dengue guidelines also recommend against routine platelet transfusion unless there is active bleeding [17]. Instead, the focus should be on close clinical monitoring, hydration, and identifying warning signs that genuinely indicate a risk of severe disease [18]. The misperception that platelet counts alone predict bleeding risk contributes to unnecessary admissions and transfusions, leading to increased healthcare costs and hospital congestion.

The burden of unnecessary hospital admissions during dengue outbreaks has serious implications for healthcare infrastructure [19]. Our study found that the median length of hospital stay was only three days, with most patients recovering without complications. This suggests that many of these admissions could have been managed on an outpatient basis. In our study, a notable finding was that 28% of the patients remained afebrile for over 72 hours despite a progressive decline in platelet count, indicating fever resolution independent of thrombocytopenia. Another study showed that outpatient management of dengue, using structured monitoring and home-based care, resulted in similar clinical outcomes while significantly reducing hospital congestion [20]. Implementing such strategies could free up hospital beds for critically ill patients and prevent the unnecessary strain on resources.

Public perception of disease severity is often influenced by media reports, which tend to emphasize severe or fatal cases while downplaying the reality that the majority of cases are mild and self-limiting [21]. This widespread fear leads to unnecessary hospital visits and admissions, further exacerbating hospital overcrowding. To address this issue, public health campaigns should focus on evidence-based messaging that educates the public about dengue’s natural course, early warning signs that warrant medical attention, and the safety of outpatient management in most cases. Collaboration between health authorities and media outlets is crucial to ensuring responsible reporting that balances awareness with accuracy [22].

Our study supports a growing consensus that dengue management should move away from hospitalization based on platelet count thresholds and instead focus on clinically significant warning signs. Key indicators for hospital admission should include persistent vomiting, severe abdominal pain, signs of plasma leakage (e.g., pleural effusion, ascites), significant mucosal bleeding, or hemodynamic instability [23].

There are several strengths and limitations in this study. A key strength of this study is its detailed analysis of clinical and laboratory trends in dengue patients, providing a comprehensive understanding of disease progression. The inclusion of real-time platelet and hematocrit monitoring adds valuable data on critical disease phases, and the study’s findings align with global research advocating for rational dengue management. In addition, the study contributes to the growing evidence that platelet count alone should not dictate hospital admission or transfusion decisions. However, several limitations must be acknowledged. The study was conducted at a single center, which may limit the generalizability of the findings to broader populations. In addition, the relatively small sample size, particularly in severe dengue cases, restricts the ability to draw firm conclusions about high-risk subgroups. The lack of long-term follow-up also prevents the assessment of post-discharge outcomes. Moreover, potential external factors such as variations in clinical management protocols and differences in patient compliance could have influenced outcomes but were not controlled for in this study. Future research should involve multi-center studies with larger and more diverse populations to validate these findings and explore structured outpatient monitoring strategies for dengue management.

## Conclusions

This study provides valuable insights into the clinical course of DF, highlighting key trends in fever resolution, thrombocytopenia, and hematocrit fluctuations, which could help refine management protocols and reduce unnecessary hospital admissions. Our findings reinforce the notion that routine hospital admissions based solely on platelet counts may not be necessary, as the majority of patients recovered without complications despite significant thrombocytopenia. The low incidence of bleeding manifestations, even in cases of severe thrombocytopenia, suggests that platelet function and endothelial integrity may play a more critical role in hemorrhagic risk than absolute platelet count alone.

These results have direct implications for clinical practice. Hospitals and clinicians could consider revising admission protocols and triage criteria to focus more on clinical signs of plasma leakage and bleeding rather than low platelet counts in isolation. This approach may support more efficient use of hospital resources, reduce fear-driven admissions, and minimize unnecessary platelet transfusions. Furthermore, integrating these findings into public health education campaigns could help improve community awareness, decrease anxiety related to dengue management, and alleviate the burden on healthcare systems during outbreaks.
